# No evidence for an association between alcohol consumption and Multiple Sclerosis risk: a UK Biobank study

**DOI:** 10.1038/s41598-022-26409-2

**Published:** 2022-12-22

**Authors:** Sapir Dreyer-Alster, Anat Achiron, Gavin Giovannoni, Benjamin M. Jacobs, Ruth Dobson

**Affiliations:** 1 Multiple Sclerosis Center, Sheba Medical Center, Ramat-Gann, Israel; 2 Preventive Neurology Unit, Wolfson Institute of Population Health, Queen Mary University London, Charterhouse Square, London, EC1M 6BQ UK; 3 Department of Neurology, Royal London Hospital, London, UK; 4 Blizard Institute, Queen Mary University London, London, UK

**Keywords:** Multiple sclerosis, Multiple sclerosis

## Abstract

Multiple Sclerosis (MS) has been linked to a variety of environmental risk factors, including smoking, Epstein-Barr Virus infection, and childhood obesity. There is some evidence to support a relationship between alcohol consumption and MS risk, but this finding has been inconsistent across cohorts. A protective link between alcohol consumption and MS risk is seen in Swedish and Danish cohorts, however evidence from other cohorts and mendelian randomisation studies have failed to support this relationship. We assessed the relationship between alcohol consumption (never vs. ever drinking) and MS in 409,228 individuals (2100 with MS) from UK Biobank (UKB). We used multivariable logistic regression models adjusted for age and sex. To determine whether there was evidence of statistical interaction between alcohol consumption and HLA-DRB1*15:01 genotype, we calculated interaction on the additive and multiplicative scales. We analysed data from 2100 individuals with MS (72.3% female, median age at recruitment 56) and 407,128 controls (53.9% female, median age at recruitment 58). We found no evidence for an association between alcohol consumption and MS risk (OR = 1.12, 95% CI 0.61–2.08, p = 0.314). As expected, the HLA-DRB1*15:01 allele was strongly associated with MS risk (OR = 2.72, 95% CI 2.72–2.72, p < 2 × 10^−16^). We found no evidence of statistical interaction between non-drinking and MS risk on either the multiplicative scale (p = 0.8) or on the additive scale (Attributable Proportion = 0.03, 95% CI − 0.43–0.29, P = 0.45). Empirical power calculations indicated reasonable statistical power (85%) to detect a protective effect of alcohol consumption of Relative Risk ≤ 0.7. We were thus unable to replicate findings from other cohorts within UK Biobank. The inconsistent association seen between studies may reflect limited statistical power to detect a weak effect, differences in population characteristics, or the lack of a true causal association.

## Introduction

Multiple Sclerosis (MS) is an autoimmune disorder of the central nervous system characterised by episodes of inflammatory demyelination superimposed on gradual neuroaxonal loss. MS is a prototypic complex disease in that there is both a heritable (genetic) and an environmental component to susceptibility^1^. A number of environmental factors associated with MS development have been discovered through large case–control and cohort studies, including infectious mononucleosis, obesity during adolescence, cigarette smoking, and low serum vitamin D^2^. A protective link between alcohol consumption and MS risk has been demonstrated in Swedish^3,4^ and Danish^5^ cohorts, but evidence from other cohorts^6^ and mendelian randomisation studies^7,8^ have failed to support this relationship.

To assess the relationship between alcohol consumption and MS risk, Hedstrom et al. analysed data from the Swedish Epidemiological Investigation of Multiple Sclerosis (EIMS). Their analysis included 2059 people with MS, and 2887 controls matched for age, sex and residential area. Alcohol consumption was categorized into drinkers versus non-drinkers, with additional analyses performed using specific alcohol consumption cut-offs (low, moderate and high consumption). Using logistic regression, the authors found a dose-dependent inverse association between alcohol consumption and MS risk (p for trend < 0.001), with a 20% risk reduction for MS among alcohol consumers compared with never-consumers (OR 0.8, 95% CI 0.7–0.9). This risk reduction was more prominent among ever smokers and among individuals carrying the HLA-DRB1*15:01 allele. The authors concluded that alcohol consumption may have a protective effect against MS, and that this protective effect may be more pronounced in individuals carrying the risk-increasing HLA allele, DRB1*15:01.

In order to determine whether the association between alcohol consumption and lower risk of MS could be replicated in a UK population, we assessed the relationship between alcohol consumption and MS in UK Biobank (UKB), a longitudinal cohort study of ~ 500,000 adults living in the UK.

## Methods

### Population

UK Biobank (UKB) is a longitudinal study of > 500,000 predominantly healthy adults based in the UK. Participants aged 40–69 were recruited between 2006 and 2010 at a range of UK centres^9^. Participants underwent a baseline visit at a UKB assessment centre where they were asked a series of questions covering demographic information, lifestyle, and medical conditions, covering a broad range of exposures and outcomes. The emphasis within UKB is on understanding the causes of age-related common diseases such as obesity, hypertension and diabetes^9^, however it remains an invaluable resource across health and chronic disease, including MS. Linked electronic healthcare records (EHR) are available for most participants in UKB, allowing researchers to integrate data from primary care attendances, secondary care, and other sources into analyses.

Within UKB, participants were genotyped using one of two genotyping chips^9^. HLA alleles were imputed from genotype array data by UK Biobank using HLA*IMP2, which generates a probabilistic dosage for the number of each HLA allele carried by each participant. For each individual, we determined the number of DRB1*15:01 alleles using probabilistic dosages (p < 0.7 = 0 alleles, p > 1.4 two alleles, 0.7 < p < 1.4 = one allele). For the primary analyses, we categorised individuals as carriers of DRB1*15:01 vs non-carriers, i.e. stipulating a dominant genetic effect. In secondary analysis we considered an additive genetic model.

From the > 500,000 individuals enrolled in UKB, we excluded participants who had asked to be removed from the study, those without high-quality genetic data (i.e. no data or > 10% missing genotypes), and individuals of non-European genetic ancestry.

### Definitions of exposures and outcomes

We defined Multiple Sclerosis cases using the ‘source of first report of G35’ UKB data field (data field ID 131043). This field is a composite outcome derived by UKB which uses data from primary care, secondary care, participant self-report, and death records. We identified individuals as MS cases if they had an MS diagnostic code in any of these data sources. In secondary sensitivity analyses, we restricted the analysis to individuals with more than one source of evidence, which diminishes sensitivity but increases the specificity of case definition. We have previously described the characteristics of this population and validated the accuracy of MS diagnoses in UKB^10^.

We used self-reported semi-quantitative alcohol consumption in order to define alcohol status (UKB field ID 1158). Participants were asked at baseline ‘About how often do you drink alcohol?’ They were prompted to give an average estimate for the preceding year if their alcohol consumption was highly variable. Possible answers were ‘daily or almost daily’, ‘3–4 times a week’, ‘once or twice a week’, ‘one to three times a month’, ‘special occasions only’, ‘never’, and ‘prefer not to answer’. Participants who answered ‘never’ were asked ‘did you previously drink alcohol?’. Individuals who indicated ‘no’ were classified as never-drinkers, and participants who indicate previous or current drinking were classified as ‘ever-drinkers’. This dichotomised variable was derived by UKB (UKB field ID 20117). Individuals with no data or who responded ‘prefer not to answer’ to both questions were excluded from downstream analysis. The validity of self-reported alcohol consumption in UKB has been demonstrated previously by showing dose–response relationships between self-reported intake, alcohol-related hospitalisation, and alcohol-related deaths^11^.

### Statistical analysis

Categorical data are presented as counts and percentages. Continuous variables are presented as median and interquartile range. Associations between alcohol drinking and MS risk were evaluated using multivariable logistic regression models adjusted for age at recruitment and sex. In secondary sensitivity analyses, we also adjusted for Townsend deprivation index, an area-level measure of socio-economic status reflecting the average deprivation level for the vicinity of the participant’s home address. Given the correlation between alcohol consumption and two established risk factors for MS – smoking (UKB field ID 20116) and early life BMI (UKB field ID 1687) – we also evaluated models adjusting for these covariates.

For the primary analyses we used data from the whole European-ancestry population after exclusion of missing/low-quality genotype or phenotype data (i.e. n = 409,580). In secondary sensitivity analyses, we repeated these association tests in a matched case–control population whereby each case was matched on age at recruitment (year) and sex to 10 controls. Matched case–control association tests were adjusted for the same covariates. Statistical interaction between alcohol drinking status and HLA-DRB1*15:01 genotype was assessed on the additive and multiplicative scales. Multiplicative interaction was assessed by incorporating an interaction term – alcohol drinking x DRB1*15:01 genotype – into the logistic models, and comparing the fit of this model to a model without the interaction term using a likelihood ratio test. Additive interaction was evaluated using the methods described by Knol et al^12^ and implemented similar to our previous work on MS in UKB^10^. Briefly, we calculated the attributable proportion (AP) due to interaction using logistic models, and determined 95% confidence intervals and empirical P values for the null hypothesis that AP = 0 by using 1000 bootstrap replicates, resampling the data with replacement 1000 times.

All analyses were conducted using R version 3.6.1 with the Queen Mary University of London Apocrita High Performance Computing (HPC) cluster. All code used for this analysis is available at https://github.com/benjacobs123456/UKB_alcohol_MS.

### Ethical approval

This research was undertaken under UK Biobank’s existing ethical approval (REC approval 11/NW/0382; North West Multi-Centre Research Ethics Committee).

### Declarations

This research was conducted using the UK Biobank resource under application 43101. All research followed the relevant regulations. Informed consent was obtained by UK Biobank for all participants. Participants who had withdrawn consent following initial enrolment were excluded from the analysis.

## Results

From an initial population of 502,486 individuals (2498 with MS, 0.5%), we excluded participants who had withdrawn consent (n = 26), those with low-quality genetic data or of non-European genetic ancestry (n = 92,880), and those with missing alcohol survey data (n = 352). This resulted in a final population of 409,228 individuals (2100 with MS, 0.51%).

Included MS cases showed the expected female predominance (n = 1519 women, 72.3%), with a median age of recruitment of 56 (IQR 13), a median age at MS report of 44.5 (IQR 17.0), and a median lag of 9.4 years from first report of MS to enrolment in UK Biobank. Controls were more evenly balanced in terms of gender (n = 219,645 women, 53.9%), but were of a similar age at recruitment (58, IQR 12). The prevalence of MS in UKB is slightly higher than contemporary estimates for the prevalence of MS in the UK in this age category^13^.

**Figure 1:**
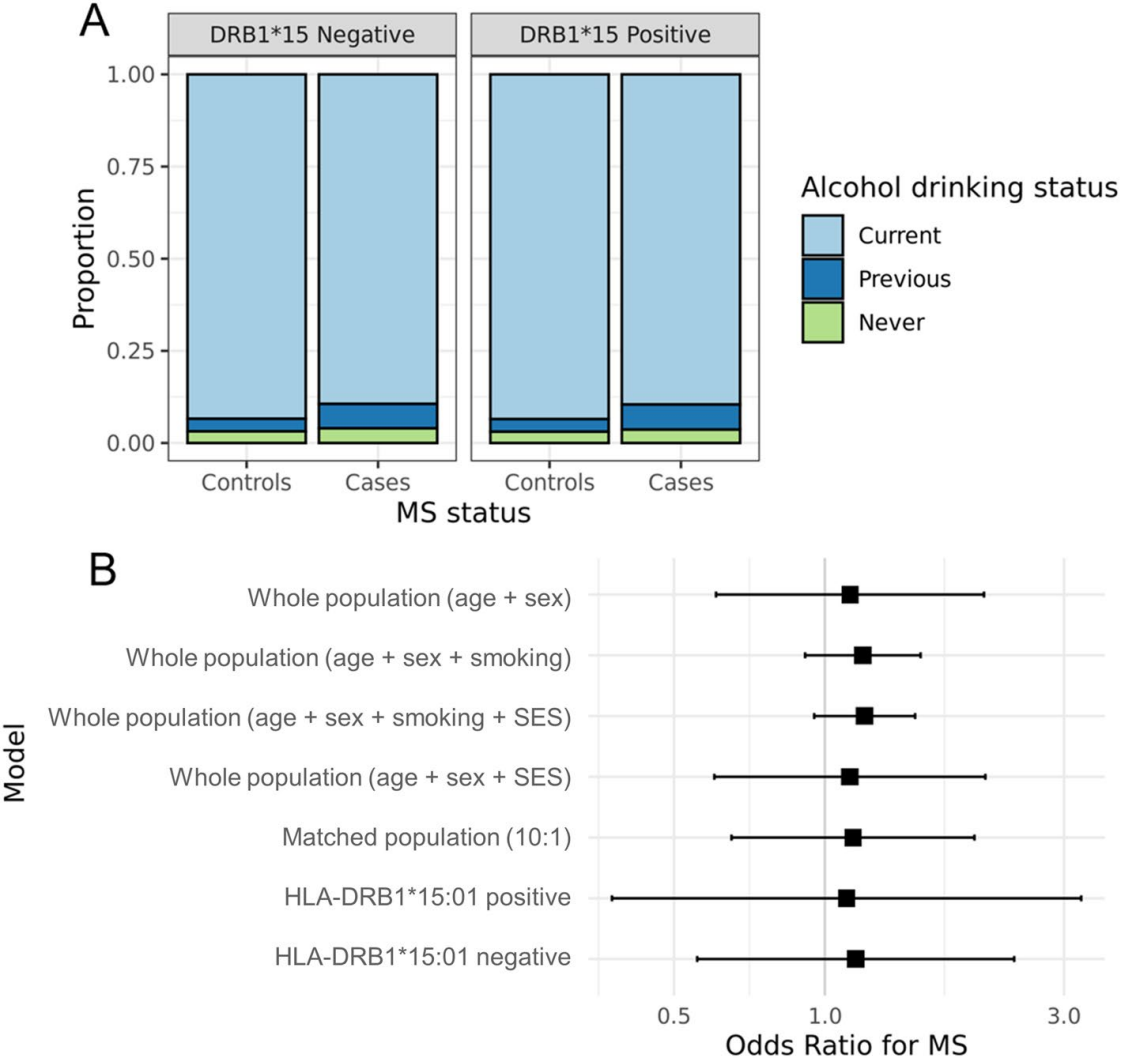
(**A**)—Alcohol drinker status for people with MS and controls in UK Biobank, divided by DRB1*15:01 negative and positive individuals. The proportion of ‘never-drinkers’ was similar between the MS and control groups. (**B**)—Forest plot depicting odds ratios for MS given lifelong abstinence from alcohol in UKB. Each point represents the OR from a multivariable logistic regression model with its 95% confidence interval. Note that the x axis is on the log10 scale. Each estimate is derived from a different model, with a variety of sensitivity analyses shown. These analyses include use of different confounding covariates, a matched nested case–control population, and a stratified analysis (by HLA-DRB1*15:01 genotype).

In total, 80 people with MS (3.8%) and 12,781 controls (3.1%) indicated they had never drunk alcohol (Fig. 1). Lifelong abstinence from alcohol was not associated with MS status in a multivariable model adjusting for age and sex (OR = 1.12, 95% CI 0.61–2.08, p = 0.314; Fig. 1).

We then explored the hypothesis that alcohol consumption is likely to correlate with MS risk factors, including BMI, cigarette smoking, and socio-economic deprivation, which could confound these observations,. In UKB, we found that never-drinkers tended to have higher Townsend deprivation index scores, indicating greater deprivation among never drinkers (univariable *t* test p < 0.0001). We found no association with childhood body size aged 10, a reasonable proxy for childhood obesity previously shown to correlate with MS risk (p = 0.8). Never-drinkers were more likely to be never-smokers (79.5% of never-drinkers reported being never-smokers, in contrast to 53.8% of ever-drinkers, p < 0.0001).

We then performed sensitivity analyses adjusting for these additional confounders (Fig. 1). Adjustment for Townsend deprivation index did not alter the results (OR = 1.12, 95% CI 0.6–2.09, p = 0.318). Similarly, adjustment for smoking status did not have an appreciable impact on the finding of no association between alcohol drinking and MS risk (OR = 1.19, 95% CI 0.91–1.55, p = 0.135). Controlling for both Townsend deprivation and smoking status in a combined model also had minimal impact on this finding (OR = 1.2, 95% CI 0.95–1.51, p = 0.118). Four individuals reported starting smoking after their MS diagnosis. Exclusion of these individuals did not affect the impact of controlling for smoking status. Finally, we created a nested matched population by matching 10 controls on age at recruitment and sex for each case. Within this nested population, we again found no evidence to support a relationship between never-drinking and MS risk (OR = 1.14, 95% CI 0.65–1.99, p = 0.285).

As expected given the strong association of HLA-DRB1*15:01 with MS risk, we identified at least one copy of the HLA-DRB1*15:01 allele in 26.8% of controls (n = 108,917) and 49.7% of MS cases (n = 1044), yielding an odds ratio of 2.7 for MS given the presence of HLA-DRB1*15:01 (OR = 2.72, 95% CI 2.72–2.72, p < 2 × 10^–16^; model adjusted for age, sex, and genetical Principal Components 1–4; PCs).

We tested for statistical interaction on the odds ratio scale between alcohol consumption and HLA-DRB1*15:01 genotype. We found no evidence for such an interaction in a multivariable model adjusting for age, sex, and PCs 1–4 (OR_interaction_ = 0.94, 95% CI 0.2–4.31, p = 0.778). We observed a similar null result when using just the 1st genetic principal component, with no principal components included in the model, with age and sex removed from the model, and when considering DRB1*15:01 as a quantitative variable (i.e. zero, one, or two alleles). We next stratified the population by HLA-DRB1*15:01 carrier status and tested for association between alcohol drinking and MS separately in the DRB1*15:01-negative and positive populations. The effect estimates were highly similar between these two strata, providing further evidence against interaction between HLA-DRB1*15:01 genotype and alcohol drinking.

Next, we tested for interaction on the additive scale, which can exist in the absence of interaction on the multiplicative (odds ratio) scale. We found no evidence of an additive interaction between never drinking and DRB1*15:01 influencing MS risk (AP_Interaction_ 0.03, 95% CI − 0.43 to 0.29, P = 0.45).

The lack of association between alcohol consumption and MS risk that we observed in this dataset could be purely due to lack of statistical power. To evaluate this possibility, we performed post hoc empirical power calculations. Assuming a total population of 409,228, of which 2100 are MS cases, and of which 12,861 do not drink alcohol, we simulated datasets under a range of plausible relative risks (i.e. relative risk for MS given lifelong abstinence from alcohol). The results of these suggest we would have reasonable power to detect a protective effect of alcohol on MS risk of an equivalent magnitude to that observed in previous observational studies (85% power for a Relative Risk of 0.7; 73% power for RR 0.75, 52% power for RR 0.8) given the rates of never-drinking and MS seen in UKB.

## Discussion

Alcohol consumption has been associated with a lower risk of MS in the Swedish case–control EIMS cohort. Here, we attempted to replicate this finding in UK Biobank. We found no evidence of a protective relationship between alcohol consumption and MS. We observed a similar proportion of never-drinkers among MS cases and controls, considering both the entire population and a nested case–control population matched 10:1 on age and sex. Controlling for age, sex, and socio-economic status did not affect these results. It must be noted that the prevalence of never drinking differed substantially between the populations; In the Swedish data, rates of never drinking were as high as 30.4% in MS cases and 27.1% in controls, compared with 4.3% and 4.4% respectively in the British data.

The disparity between UKB and EIMS may reflect differences in exposure and outcome recording. Although in both populations alcohol consumption was assessed in a cross-sectional manner, the EIMS data are likely to be more accurate as the participants were surveyed closer to diagnosis, and so their historical alcohol consumption (prior to diagnosis) is likely to bear a closer relationship to their current alcohol consumption. The cross-sectional nature of the UKB data is a clear limitation to our study – although we can be relatively confident that never-drinkers have never drunk alcohol, it is not possible to accurately examine alcohol consumption prior to diagnosis in UKB. Reassuringly, 83% of both the MS and control populations answered that their alcohol consumption was similar to or lower than their alcohol consumption 10 years prior (UKB field ID 1628). Furthermore, binary definitions as used in both studies cannot capture behaviour change or a time dependent influence of consumption on MS risk.

MS diagnoses were derived from electronic healthcare records in UKB, and so may be less accurate than the clinician-verified diagnoses in EIMS. It is unclear whether differences in reported alcohol consumption purely reflect cultural differences in drinking contribute to this disparity. Data from the World Health Organisation (WHO) support the view that alcohol consumption differs markedly between the UK and Sweden: the WHO estimates that the per-capita annual consumption of alcohol among adults is ~ 2.7 L (of pure alcohol) higher in the UK than in Sweden^14^.

It remains possible that the lack of association we observe is due to lack of statistical power. The lower rate of never-drinking in the UKB population diminished the power, and so it is plausible that we would miss a weak protective effect of alcohol in this study – as our power calculations demonstrate, a weak protective effect (RR 0.8) would only be detectable with only 52% power at an alpha of 0.05. However, we estimate reasonable power (85%) to detect a stronger effect of the magnitude similar to that reported in a Danish cohort (OR < 0.7)^5^.

A further limitation of UKB is that participation in the study is non-random, and the study population is therefore not necessarily representative of the broader population^15^. It is known that the UKB population is enriched for healthy individuals with lower rates of smoking, alcohol consumption, and obesity compared with the general population^16^. This could plausibly affect our results through collider bias. Further population-based studies will be required to definitively answer this question.

Despite these limitations, it is notable that the relationship between alcohol and MS risk is heterogeneous between studies. Here we report that in a large UK population (UK Biobank), there is a lack of evidence to support the hypothesis that alcohol protects against MS in a manner that is modified by HLA-DRB1*15:01 genotype. Further research in population-based studies and more diverse cohorts is required to clarify whether such a relationship is causal or has arisen due to bias.

## Data Availability

All code used to perform the analysis is available at https://github.com/benjacobs123456/UKB_alcohol_MS. UK Biobank data can be obtained by applying here: https://www.ukbiobank.ac.uk/.
